# Corneal bee sting: improvement in the acute stage in the absence of treatment

**DOI:** 10.11604/pamj.2020.37.54.20267

**Published:** 2020-09-14

**Authors:** Michael Carl Chen, Veniamin Melnychuk

**Affiliations:** 1 Tenwek Hospital, Bomet, Bomet County, Kenya,; 2 Penn State Eye Center, Hershey, Pennsylvania,; 3 Penn State College of Medicine, Hershey, Pennsylvania

**Keywords:** Bee sting, cornea, eye, ophthalmology

## Abstract

Bee sting injuries to the eye are relatively uncommon. The outcomes of corneal bee sting injuries are highly variable, and there is no consensus on the management at the time of initial presentation. We report a case of a 17-year-old male with a corneal bee sting injury that significantly improved in the acute stage without any treatment, which to the best of our knowledge is the first report in the literature documenting the natural history of a corneal bee sting injury in the acute stage.

## Introduction

Patients with a bee sting injury to the cornea usually present with pain and decreased vision associated with clinical findings of conjunctival injection, corneal infiltration, corneal edema, and anterior chamber inflammation [1-4]. The outcomes of corneal bee sting injuries are highly variable, with outcomes ranging from mild loss of vision to more serious sequelae requiring surgical intervention, such as corneal decompensation or scarring, cataract formation, and glaucoma [2]. There is no consensus on the management at the time of initial presentation, with recommendations consisting of medical management alone to recommendations that also include the surgical removal of the stinger [2-4]. We report a case of a corneal bee sting injury that significantly improved in the acute stage without any treatment.

## Patient and observation

**Figure 1:**
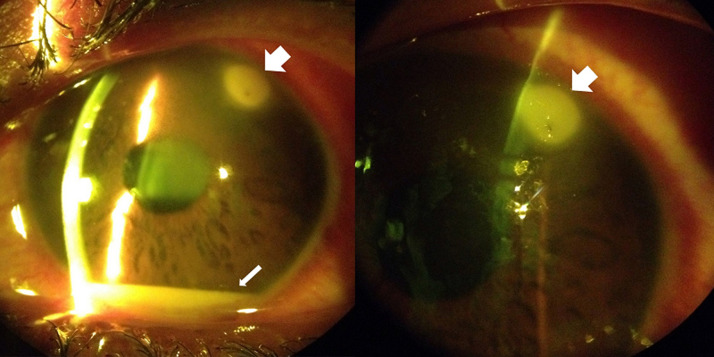
slit lamp photos one day post injury, showing the stinger with associated infiltrate (thick arrow) and hypopyon (thin arrow)

**Figure 2:**
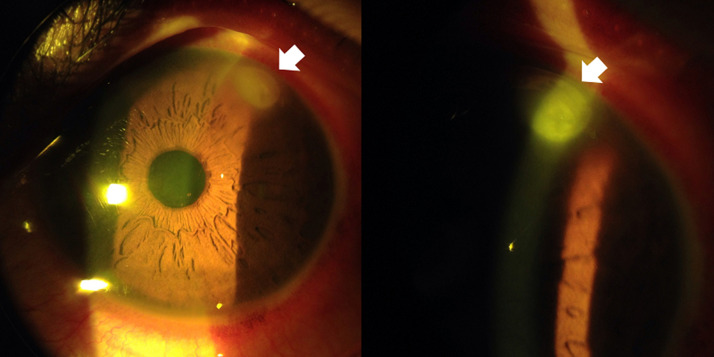
slit lamp photos four days post injury, showing the improved infiltrate (thick arrow) and resolution of the hypopyon

A 17-year-old male with no prior ocular history presented to Tenwek Hospital in Bomet, Kenya with redness, pain, photophobia, and decreased vision in the left eye. He reported his eye being stung by a bee the day before while working in the fields. His visual acuity was 6/6 RE and 6/9 LE. Intraocular pressure was 9 mm Hg RE and 10 mm Hg LE. Slit lamp examination of the left eye revealed grade 2 diffuse conjunctival injection with ciliary flush, a stinger associated with a 1.7 mm diameter infiltrate in the superotemporal cornea, moderate corneal stromal edema, grade 4 cell with fibrin, and a 1 mm hypopyon (Figure 1). Attempt was made to remove the stinger at the slit lamp under topical anesthesia with jeweler forceps without success. He was prescribed topical prednisolone, ciprofloxacin, econazole, chloramphenicol, and atropine. Three days later the patient returned for follow-up with improvement in symptoms. His visual acuity in the left eye was 6/6. Intraocular pressure was 10 mm Hg. The infiltrate had decreased in density and dimension to 1.5 mm diameter. There was improved corneal stromal edema. The anterior chamber cell had decreased to grade 1, and the hypopyon had resolved. The stinger was no longer visible, as likely it had been pushed deeper into the cornea stroma on failed attempted removal and was obscured by the remaining infiltrate (Figure 2). To the surprise of the author (M.C.), the patient had not obtained or used any eye drops that were prescribed at the previous visit. The patient was encouraged to follow through with the treatment recommended at the previous visit and to follow up in one week, but he did not return and was lost to follow-up.

## Discussion

The outcomes of corneal bee and wasp stings are highly variable and there is no consensus on the management at acute presentation. While some reports describe the surgical removal of the stinger [2], in the case that a stinger is not easily accessible, not in the visual axis, and is not associated with significant infiltration, close observation and medical management with topical/oral antibiotics, corticosteroids, and cycloplegics has also been described [4]. While there has been a description of a retained stinger inducing chronic keratouveitis [5], there has also been a description where a intralenticular stinger was retained for up to 28 years without any inflammatory complications [6]. The wide range of response may at least be in part due to the differences in venom composition among the different species of bees and wasps [7]. In such cases where the stinger was left retained without complications, it is postulated that after the venom becomes neutralized that the retained stinger becomes inert [8].

Rai *et al*. reported a case of a corneal bee sting injury where corticosteroids were withheld due to concern for infection, and significant improvement occurred after 5 days with antibiotics alone, in the presence of a retained stinger [3]. While the authors postulated that the underlying process was primarily infectious, the presence of infection was not confirmed, as cultures and PCR were not performed. In this present case, improvement in inflammation occurred within 3 days with a retained stinger and in the absence of treatment, supporting the theory that at least in some cases, the acute inflammatory insult is short-lived and the inflammation can improve without intervention. The potential long-term complications reported in the literature, such as corneal decompensation or scarring, cataract formation, and glaucoma [2], are unknown in this case, as unfortunately the patient was lost to follow-up.

## Conclusion

To the best of our knowledge, this is the first report in the literature documenting the natural history of a corneal bee sting injury in the acute stage in the absence of treatment. While the outcomes of corneal bee sting injuries are highly variable and the outcomes of this case cannot be generalizable to other cases of corneal bee sting injuries, this case demonstrates that at least in some instances the acute inflammatory response is transient and can improve in the absence of treatment.
